# MEDIATION OF THE NUTRITIONAL STATUS BETWEEN THE NUMBER OF TEETH AND DEPRESSION AMONG OLDER ADULTS IN WESTERN CHINA

**DOI:** 10.1093/geroni/igae098.0088

**Published:** 2024-12-31

**Authors:** Xin Tian, Yuexia Hu, Yuqing Xie, Xin Xia, Yanyan Wang

**Affiliations:** Sichuan University, Chengdu, China (People’s Republic); Sichuan university, Chengdu, Sichuan, China (People’s Republic); Sichuan university, Chengdu, Sichuan, China (People’s Republic); Center of Gerontology and Geriatrics, West China Hospital, Sichuan University/ National Clinical Research Center for Geriatrics, West China Hospital, Sichuan University, Chengdu, Sichuan, China (People’s Republic); West China School of Nursing, Sichuan University, Chengdu, Sichuan, China (People’s Republic)

## Abstract

Depression is a common mental disorder. Studies have shown that depression is associated with the number of teeth, but underlying mechanisms deserve further research. Our study aimed to explore the mediating effect of nutritional status between the number of teeth and depression. We analyzed cross-sectional baseline data from the 2018 West China Health and Aging Trend study (WCHAT), including 6632 multi-ethnic adults aged 50-95. The survey tool for depressive symptoms is 15-item Geriatric Depression Scale (DGS-15), and nutritional status was evaluated through Mini Nutritional Assessment-Short Form (MNA-SF). We conducted a multiple linear regression to estimate relationships between the number of teeth, nutritional status, and depression. Mediation models and pathway analysis were constructed to figure out the mediating role of nutritional status. Of 6632 participants, the depressive symptoms proportion was 17.3%. Multiple linear regression proved a total association between the number of teeth and depression (β = -0.148; 95% confidence interval: -0.218 to -0.078), the associations remained significant after adjusting MNA-SF scores. Mediation analysis verified nutritional status partially mediates the associations between the number of teeth and depression (indirect effect estimate = -0.059, 95% CI: -0.076 to -0.044; direct effect estimate = -0.088, 95% CI: -0.155 to -0.017). Structural equation model framework pathway analysis confirmed the association between the number of teeth, nutritional status, and depression. Our data suggested that nutritional status partially mediated the relationship between the number of teeth and depression, early identification of nutritional status and emphasis on oral hygiene are necessary to mitigate the risk of depression.

